# Endocannabinoids Mediate Muscarinic Acetylcholine Receptor-Dependent Long-Term Depression in the Adult Medial Prefrontal Cortex

**DOI:** 10.3389/fncel.2015.00457

**Published:** 2015-12-01

**Authors:** Henry G. S. Martin, Axel Bernabeu, Olivier Lassalle, Clément Bouille, Corinne Beurrier, Anne-Laure Pelissier-Alicot, Olivier J. Manzoni

**Affiliations:** ^1^Aix-Marseille UniversitéMarseille, France; ^2^Institut de Neurobiologie de la Méditerranée UMR_S 901Marseille, France; ^3^INMED UMR_S 901Marseille, France; ^4^APHM, CHU Conception, Service de PsychiatrieMarseille, France; ^5^Centre National de la Recherche Scientifique, Institut de Biologie du Développement de Marseille UMR 7288Marseille, France; ^6^APHM, CHU Timone Adultes, Service de Médecine LégaleMarseille, France

**Keywords:** M_1_ muscarinic acetylcholine receptor, CB_1_ receptor, prefrontal cortex, long-term depression, eCB system, synaptic plasticity

## Abstract

Cholinergic inputs into the prefrontal cortex (PFC) are associated with attention and cognition; however there is evidence that acetylcholine also has a role in PFC dependent learning and memory. Muscarinic acetylcholine receptors (mAChR) in the PFC can induce synaptic plasticity, but the underlying mechanisms remain either opaque or unresolved. We have characterized a form of mAChR mediated long-term depression (LTD) at glutamatergic synapses of layer 5 principal neurons in the adult medial PFC. This mAChR LTD is induced with the mAChR agonist carbachol and inhibited by selective M_1_ mAChR antagonists. In contrast to other cortical regions, we find that this M_1_ mAChR mediated LTD is coupled to endogenous cannabinoid (eCB) signaling. Inhibition of the principal eCB CB_1_ receptor blocked carbachol induced LTD in both rats and mice. Furthermore, when challenged with a sub-threshold carbachol application, LTD was induced in slices pretreated with the monoacylglycerol lipase (MAGL) inhibitor JZL184, suggesting that the eCB 2-arachidonylglyerol (2-AG) mediates M_1_ mAChR LTD. Yet, when endogenous acetylcholine was released from local cholinergic afferents in the PFC using optogenetics, it failed to trigger eCB-LTD. However coupling patterned optical and electrical stimulation to generate local synaptic signaling allowed the reliable induction of LTD. The light—electrical pairing induced LTD was M_1_ mAChR and CB_1_ receptor mediated. This shows for the first time that connecting excitatory synaptic activity with coincident endogenously released acetylcholine controls synaptic gain via eCB signaling. Together these results shed new light on the mechanisms of synaptic plasticity in the adult PFC and expand on the actions of endogenous cholinergic signaling.

## Introduction

Acetylcholine is essential to learning and memory processes as well as synaptic plasticity in the CNS (Purves et al., 2001). The plethora of acetycholine physiological functions are underlined by the presence of both nicotinic ion channel receptors (nAChR) and muscarinic G-protein coupled receptors (mAChR).

In the perirhinal cortex, mAChRs of the M_1_ subtype underlie synaptic plasticity and recognition memory (Massey et al., 2001; Warburton et al., 2003). Likewise the prefrontal cortex (PFC) is sensitive to regulation by M_1_ mAChRs, where in concert with nAChRs it modulates attention (Hasselmo and Sarter, 2011; Klinkenberg et al., 2011; Bloem et al., 2014). Furthermore, both pharmacological and genetic disruption of M_1_ mAChR signaling has been linked to deficits in learning and memory of PFC dependent tasks (Anagnostaras et al., 2003; Carballo-Márquez et al., 2007; Barker and Warburton, 2008). Recent studies have identified an activity-dependent long-term depression (LTD) in deep layers of the rat medial PFC (mPFC) mediated by the M_1_ mAChR that may be relevant neurological disorders (Huang and Hsu, 2010; Caruana et al., 2011; Ghoshal et al., 2015). However, despite the importance of M_1_ mAChRs in the behavioral function and neuropathology of the mPFC, the expression mechanism of this LTD is unknown.

One of the major forms of LTD found at most excitatory circuits in the CNS is mediated by the endogenous cannabinoid (eCB) system (Heifets and Castillo, 2009; Katona and Freund, 2012). Retrograde eCB signaling typically requires the synaptic activation of postsynaptic G-coupled group I metabotropic glutamate receptors (mGluR) positively coupled to the phospholipase C (PLC)/intracellular Ca^2+^ pathways. Production and release of eCBs then acts at presynaptic CB_1_ receptors (CB_1_R; Robbe et al., 2002; Heifets and Castillo, 2009; Katona and Freund, 2012). In the mPFC, strong evidence links disruption of eCB-LTD to neuropsychiatric diseases (Lafourcade et al., 2007; Kasanetz et al., 2013; Thomazeau et al., 2014). In addition to mGluR1/5, activation of other G_q/11_ coupled receptors also produce eCBs and eCB dependent synaptic plasticity (Heifets and Castillo, 2009; Katona and Freund, 2012). Notably, activation of M_1_ mAChR triggers PLC dependent eCB release and can enhance short and long-term eCB plasticity at central synapses (Kim et al., 2002; Ohno-Shosaku et al., 2003; Narushima et al., 2007; Zhao and Tzounopoulos, 2011).

Here, we tested the possibility that M_1_ mAChR LTD requires eCB signaling in the adult mPFC. We found that in both the rat and mouse mPFC blockade of the CB_1_R prevented carbachol induced M_1_ mAChR LTD. Furthermore subthreshold stimulation of mAChRs was converted to LTD when the degradation of the principal eCB 2-arachidonoylglycerol (2AG) was blocked. Finally in mice selectively expressing channelrhodopsin in cholinergic neurons, we show that light induced release of endogenous acetylcholine causes an LTD, mediated by M_1_ mAChRs and CB_1_Rs.

## Materials and Methods

All mice and rats were group housed with 12 h light/dark cycles in compliance with the European Communities Council Directive (2010/63/EU) for the Care and Use of Laboratory Animals. Project ethical committee approval was sought from the Comité éthique de Provence and all experiments were performed locally under European Union approval (agreement number: B 13-055-19). Male C57Bl/6J, ChR2:ACh mice on a C57Bl/6J genetic background and Wistar rats were used. ChR2:ACh mice were generated by crossing Choline Acetyltransferase ChAT-IRES-Cre knock-in mice (ChATcre/cre mice, stock number: 006410) with LoxP-stop-ChR2(H134R)-EYFP mice (Ai32 mice, stock number: 012569), where ChR2(H132R)-EYFP expression is restricted by a *loxP-*flanked STOP cassette (Madisen et al., 2012). All experiments were performed on adult animals; rats 3–4 months, mice 10–14 weeks.

**Figure 1:**
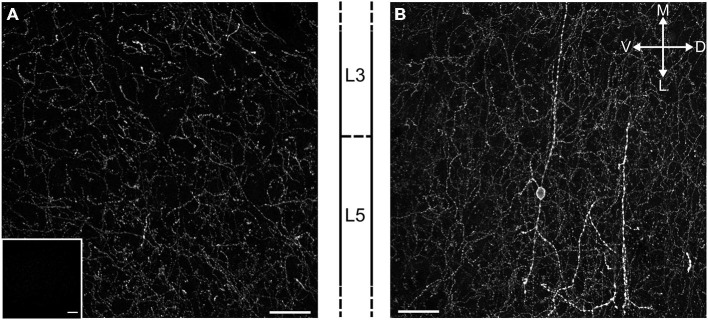
**Cholinergic terminals expressing ChR2-EYFP richly innervate the mPFC in ChR2:ACh mice. (A)** Fluorescent image of a projected confocal *z*-stack 20 μm thick from the prelimbic cortex of the ChR2:ACh mouse. Approximate cortical layer boundaries are indicated to the right. Insert: similar image from a control C57Bl/6J mouse mPFC section acquired with identical settings. **(B)** Example of a presumed bipolar local cholinergic interneuron in mPFC from ChR2:ACh mouse. Image represents projected confocal image of YFP signal from prelimbic cortex coronal section. Approximate layer boundaries are indicated to the left. Orientation markers: D, dorsal; V, ventral; M, medial; L, lateral; scale bar: 30 μm.

Medial PFC expression of channelrhodopsin 2 in ChR2:ACh mice was confirmed by confocal microscopy. Direct fluorescent microscopy of the ChR2(H134R)-EYFP fusion protein showed that cholinergic afferents richly innervate the mPFC with corresponding channelrhodpsin expression in all cortical layers (Figure 1A). Furthermore we were able to identify a sparse population of bipolar cells that expressed channelrhodpsin locally (Figure 1B). These cells situated in layer 5 project neurites perpendicular to the cortical layer boundaries into both superficial and deep layers. We hypothesize these are previously reported cholinergic interneurons (Houser et al., 1985; Van der Zee and Keijser, 2011).

### Slice Preparation

Rats and mice were anesthetized with isoflurane and decapitated according to institutional regulations. Brains were sliced (250–300 μm) in the coronal plane with a vibratome (Integraslice, Campden Instruments, Loughborough, UK) in a sucrose-based solution at 4°C (in mM: 87 NaCl, 75 sucrose, 25 glucose, 5 KCl, 21 MgCl_2_, 0.5 CaCl_2_ and 1.25 NaH_2_PO_4_). Slices were allowed to recover for 60 min at 32–35°C in artificial cerebrospinal fluid (aCSF) containing 126 mM NaCl, 2.5 mM KCl, 2.4 mM MgCl_2_, 1.2 mM CaCl_2_, 18 mM NaHCO_3_, 1.2 mM NaH_2_PO_4_ and 11 mM glucose, equilibrated with 95% O_2_/5% CO_2_. Slices were then maintained at 22 ± 2 °C until recording.

### Electrophysiology

Whole-cell patch-clamp and extra-cellular field recordings were made from layer 5 pyramidal cells of the prelimbic cortex (Lafourcade et al., 2007; Kasanetz et al., 2013; Martin et al., 2015). For recording, slices were superfused (1.5–2 ml/min) with aCSF. All experiments were performed at 32 ± 2 °C. The recording a CSF contained picrotoxin (100 μM) to block GABA_A_ receptors. To evoke synaptic currents, 100–200 μs stimuli were delivered at 0.1 Hz through an aCSF-filled glass electrode positioned dorsal to the recording electrode in layer 5. Patch-clamp recordings were performed with a potassium gluconate based intracellular solution (values mM: 143 potassium gluconate, 3 NaCl, 1 MgCl_2_, 0.3 CaCl_2_, 1 EGTA, 0.2 cAMP, 0.3 NaGTP, 2 NaATP, 10 HEPES (pH 7.25), osmolarity 290–300 mol/L). Patch pipettes had a resistance between 3 and 5 MΩ. In all experiments cells were clamped at −70 mV (without junction potential correction). During recordings holding currents, series and input resistances and the membrane time constant (τ) were monitored. If the series resistance exceeded 25 MΩ or varied by >20% during the experiment the recording was rejected. For extracellular field experiments, the recording pipette was filled with aCSF. The glutamatergic nature of the field excitatory postsynaptic potential (fEPSP) was systematically confirmed at the end of the experiments using the ionotropic glutamate receptor antagonist 6-cyano-7-nitroquinoxaline-2,3-dione (CNQX, 20 μM), that specifically blocked the synaptic component without altering the non-synaptic component (not shown). Example EPSPs and fEPSPs are single sweeps from the indicated time points, for clarity the stimulation artifact was removed from the fEPSP.

### Optogenetics

A 473 nm laser (Dragon Laser China) coupled to a 50 μm core glass silica optical fiber (ThorLabs) was positioned directly above the slice orientated at 30° approximately 350 μm from the recording electrode. Light intensity was calibrated to 2 mW at the fiber tip. At the site of recording discounting scattering a region of approximately 0.05 mm^2^ was illuminated that after power attenuation due to adsorption and scattering in the tissue was calculated as approximately 100 mW mm^−2^ (Yizhar et al., 2011). During repetitive stimulation this power intensity did not induce phototoxic effects (determined by field EPSP stability), yet was sufficient to reliably induce acetylcholine mediated LTD. Cholinergic fibers were excited by brief 5 ms pulses via a Master-8 pulse stimulator (AMPI).

A paired light EPSP protocol was developed to induce M_1_ mAChR LTD in ChR2:ACh mice. Local field EPSPs were first induced via a stimulation electrode positioned within the laser illuminated region surrounding the recording electrode. After a stable field EPSP baseline period, LTD was induced by pairing two pulses of light (50 ms interval) with a field EPSP co-incident with the second light pulse. Paired light EPSP stimulation was performed at 1 Hz for 1200 repetitions.

### Data Acquisition and Analysis

Data was recorded on a MultiClamp 700B amplifier, filtered at 2 kHz, digitized (10 kHz or 50 kHz, DigiData 1440A), collected using Clampex 10.2 and analyzed using Clampfit 10.2 (Molecular Devices, Sunnyvale, USA). Analysis of both area and amplitude of fEPSPs was performed. The magnitude of LTD was calculated 30–40 min after the end of the induction protocol as percentage of baseline responses. Resultant changes in synaptic currents were analyzed with paired Student’s *t*-tests. The paired-pulse ratio (PPR) was calculated from the peak current of two closely spaced EPSCs (50 ms), such that the PPR = Peak 2/Peak 1. Quoted PPR values are the average of 30 trials. The effect of group treatments on LTD were tested by one-way ANOVA and Holm-Sidak multiple comparisons test. N values represent individual animals. All values are given as mean ± standard error and statistical significance was set at *P < 0.05 and **P < 0.01.

### Drugs

Drugs were added at the final concentration to the recording aCSF media. Picrotoxin was from Sigma (St. Quentin Fallavier, France); VU0255035, JZL184, AMG 9810, AM251, carbachol and pirenzepine dihydrochloride from Tocris, CNQX and D-AP5 was from the National Institute of Mental Health’s Chemical Synthesis and Drug Supply Program (Rockville, MD, USA).

## Results

### M_1_ Muscarinc Acetylcholine Receptor LTD is Mediated by the CB_1_R in the Adult Rat PFC

**Figure 2:**
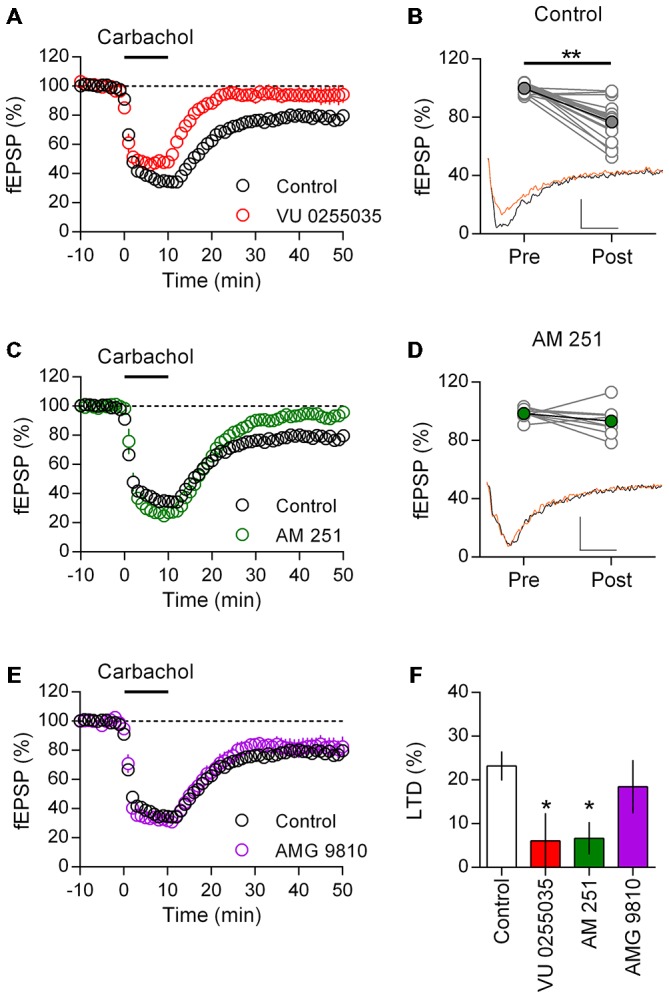
**M_1_ muscarinic acetlycholine receptor (mAChR) dependent long-term depression (LTD) is CB_1_ receptor (CB_1_R) mediated in adult rat mPFC. (A)** Time course of normalized field EPSPs during acute carbachol (10 μM, 10 min) stimulation in presence of M_1_ receptor antagonist VU0255035 (10 μM, *n* = 5; control, *n* = 17). **(B)** Individual experiments from control group (gray), pre (baseline) and post (40 min washout) carbachol treatment and in black group average (**P < 0.01). Below, example traces from baseline (black) and 40 min post (orange) carbachol treatment (scale bar: 5 ms, 0.1 mV). **(C)** Time course of normalized field EPSPs in presence of CB_1_R antagonist AM251 (4 μM, *n* = 8) during carbachol induced LTD. **(D)** Pre and post carbachol field EPSPs for individual experiments (gray) and group average (green) in presence of AM251; pre and post carbachol example traces below. **(E)** Time course of normalized field EPSPs during carbachol induced LTD in presence of transient receptor potential vanilloid type 1 (TRPV1) receptor antagonist AMG 9810 (3 μM, *n* = 9). **(F)** Summary bar chart of percent LTD 40 min after carbachol washout (*P < 0.05).

Layer 5 mPFC pyramidal neurons in adult rodents are notable for expressing a prominent eCB mediated LTD in response to direct mGluR5 activation or patterned glutamatergic stimuli (Lafourcade et al., 2007; Jung et al., 2012). This population of neurons also express M_1_ mAChRs that may couple to the same set of secondary messengers as the mGluR5 (Yamasaki et al., 2010). Given the role of acetylcholine in mPFC mediated tasks throughout life, we asked if M_1_ mAChR activation might also result in eCB-LTD in the adult rat mPFC. Recording field EPSPs from layer 5 of the mPFC, we first challenged acute brain slices with a brief (10 min, 10 μM) carbachol stimulation to test whether M_1_ mAChR LTD is expressed in these neurons (Figure 2A). As reported in juvenile rats (Huang and Hsu, 2010; Caruana et al., 2011), carbachol induced a rapid depression of responses that following washout incompletely recovered and stabilized at 76.8 ± 3.3% (*n* = 17) of the baseline response. Notably, incubating the slices in the highly selective M_1_ mAChR antagonist VU0255035 had no effect on the acute carbachol induced depression of responses, however after washout of carbachol responses recovered to baseline values (93.9 ± 6.4%, *n* = 5). We compared individual field EPSPs before and 40 min after carbachol washout in control animals (Figure 2B). Carbachol treatment resulted in a highly significant depression of field EPSP responses (*t*_(16)_ = 6.60, *P* < 0.001).

M_1_ mAChR and mGluR5 engage the same G_q/11_ class of G-proteins and may activate 2-AG synthesis and CB_1_R mediated depression of synaptic transmission (Kim et al., 2002; Katona and Freund, 2012). Therefore in the presence of the selective CB_1_R antagonist AM251, we challenged layer 5 neurons to the same brief carbachol stimulation (Figure 2C). As before carbachol induced a strong acute depression of field EPSPs, however in the presence of the CB_1_R antagonist responses returned to baseline values after carbachol washout (93.3 ± 3.7%, *n* = 8). Plotting of individual experiments indicated there was no significant difference in field EPSP responses after carbachol stimulation in the presence of AM251 (Figure 2D). Furthermore, compared to control experiments AM251 significantly interacted with the carbachol induced LTD in these neurons (two-way repeat measure ANOVA *F*_(1,23)_ = 9.43, *P* = 0.006), which was due to an inhibition of the carbachol mediated LTD (*P* < 0.001, Holm-Sidak’s multiple comparisons test).

In addition to the CB_1_R, the Transient Receptor Potential Vanilloid Type 1 (TRPV1) receptor is both expressed in the mPFC and activated by eCBs (Mezey et al., 2000). Through the mobilization of intracellular calcium stores, mAChR activation can enhance formation of the eCB anandamide leading to TRPV1 channel opening (van der Stelt et al., 2005). Therefore we asked if the TRPV1 receptor might also contribute to the induction of M_1_ mAChR LTD. Recording field EPSPs, we incubated layer 5 mPFC neurons in the TRPV1 antagonist AMG 9810 before again challenging with carbachol. However in contrast to the CB_1_R antagonist, AMG 9810 did not affect the induction of LTD (Figure 2E) and field responses remained significantly depressed compared to baseline (81.5 ± 6.1%; *t*_(8)_ = 2.78, *P* = 0.024). Furthermore, the carbachol LTD time course in the presence of AMG9810 broadly matched that of control neurons, indicating the TRPV1 receptor is not involved in M_1_ mAChR mediated LTD. Expressing the LTD as a percent depression of baseline values, we compared the carbachol effect across treatments and identified a significant effect (one-way ANOVA *F*_(3,35)_ = 3.41, *P* = 0.028; Figure 2F). Treatment with either M_1_ mAChR antagonist or CB_1_R antagonist significantly reduced the induction of LTD compared to controls (VU0255035: *P* < 0.05; AM251: *P* < 0.05; Holm-Sidak’s multiple comparisons test), however with TRPV1 receptor antagonist the percent LTD was not significantly changed.

### M_1_ mAChR LTD is Mediated by the Action of the eCB 2-AG at the Presynapse

CB_1_Rs in the CNS are principally activated by 2-arachidonoylglycerol (2-AG). In the classic model, 2-AG is synthesized in the post-synaptic neuron by diacylglycerol lipases before signaling in a retrograde direction to pre-synaptic CB_1_R which in turn depress neurotransmitter release (Heifets and Castillo, 2009). In the presynapse 2-AG is then rapidly degraded by resident lipases, principally by monoacylglycerol lipase (MAGL; Lafourcade et al., 2007; Marrs et al., 2010).

**Figure 3:**
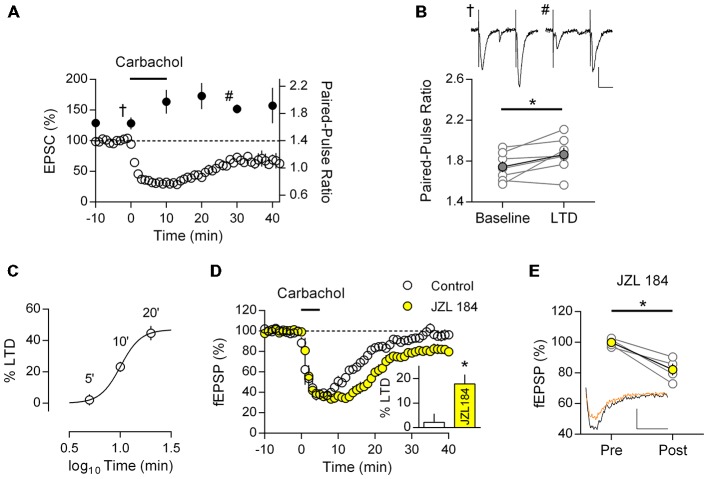
**M_1_ mAChR dependent LTD is expressed in the presynapse and is mediated by the endogenous cannabinoid (eCB) 2-AG. (A)** Time course of normalized EPSC in response to carbachol (10 μM, 10 min) from layer 5 principal neurons (*y*-axis: left; clear data points), co-plotted with paired-pulse ratio (PPR; 50 ms interval) at indicated time points (*y*-axis: right; filled data points). **(B)** Plot of PPR from individual neurons (gray), before (baseline) and 20 min after (LTD) carbachol challenge; group averages superimposed (filled data points, *n* = 7, *P < 0.05). Example traces taken at the indicated time points **(A)** during baseline (†) and after LTD (#) shown above (scale bar: 20 ms, 50 pA). **(C)** Time—response plot of percent LTD resulting from 10 μM carbachol challenge for varying time periods. **(D)** Time course of normalized field EPSPs during sub-threshold carbachol challenge (10 μM, 5 min) in presence of MAG lipase inhibitor JZL184 (1 μM, *n* = 4; control, *n* = 6). Insert: fEPSPs expressed as percent LTD 40 min after challenge. **(E)** Before-after responses from individual experiments (gray) of baseline (Pre) and 40 min after sub-threshold carbachol challenge (Post) in presence of JZL184; in yellow group average. Example traces below, pre carbachol (black) and post (orange; scale bar: 10 ms, 0.1 mV).

If M_1_ mAChR LTD follows the classic model of CB_1_R mediated LTD it is expected that the resultant synaptic depression will have a presynaptic loci. To test this prediction we recorded the PPR from patched neurons during the induction of M_1_ mAChR LTD; any change in this measure would suggest the LTD involves a presynaptic component. Similar to field EPSP recordings, we found that patch-clamped neurons also undergo a robust LTD in response to carbachol challenge (10 min, 10 μM) resulting in a sustained depression in EPSC (67.2 ± 4.8% of baseline, *n* = 7; Figure 3A). Initial responses from these neurons showed a weak paired-pulse facilitation in response to closely spaced EPSCs (1.66 ± 0.09, *n* = 9). However carbachol induced LTD resulted in sharp increase in the PPR that persisted and was concomitant to M_1_ mAChR LTD (Figure 3A). Comparing the PPR from individual neurons before and after M_1_ mAChR LTD, we found a significant increase in the PPR (Figure 3B; *t*_(6)_ = 2.519, *P* = 0.045). Such an increase in paired-pulse facilitation is consistent with a reduction in neurotransmitter release probability and the expression of M_1_ mAChR LTD in the presynapse.

To investigate the role of 2-AG in M_1_ mAChR LTD, we took advantage of the sensitivity of CB_1_R mediated LTD to perturbations in the local concentration of 2-AG. Specifically, subthreshold stimulation protocols or pathological deficits in 2-AG eCB signaling may be restored to express LTD by manipulations that augment the synaptic concentration of 2-AG (Lafourcade et al., 2007; Marrs et al., 2010; Jung et al., 2012). Thus if a nominally subthreshold M_1_ mAChR activation is sensitive to manipulation of 2-AG concentration, this suggests M_1_ mAChR LTD engages the classic eCB-LTD model. We focused on the degradation of 2-AG, since it has previously been shown to have a critical role in eCB signaling and also due to the availability of highly selective drugs targeting MAGL (Long et al., 2009).

We first challenged mPFC neurons with different time periods of carbachol stimulation to determine a time—response profile for M_1_ mAChR LTD, whilst maintaining the same concentration of carbachol (10 μM). All intervals of carbachol challenge resulted in an acute depression of field EPSPs (Figures 2, 3), however after 40 min washout responses recovered to baseline with a 5 min challenge (97.8 ± 3.5%, *n* = 6) or were progressively depressed depending on the duration of carbachol challenge (10 min: 76.8 ± 3.3%, *n* = 17; 20 min: 55.3 ± 4.6%, *n* = 3). Expressing responses as percent LTD we plotted the time—response profile for carbachol induced LTD and fitted a four-parameter logistic curve (Figure 3C, R^2^ = 0.53). The best-fit constants: LTD_MAX_ 47.0% (24.5–69.4, 95% confidence interval), EC_50_ 10.1 min (7.7–13.1, 95% confidence interval), suggest that a 20 min carbachol challenge is saturating.

Selecting the 5 min subthreshold carbachol challenge, we repeated experiments in the presence of the selective MAGL inhibitor JZL184. In both conditions carbachol induced an acute depression of field EPSPs from layer 5 neurons which was similar in both groups, however in the presence of JZL184 responses remained depressed after carbachol washout (Figure 3D). Average evoked responses 40 min after carbachol washout were significantly different between control and JZL184 treated neurons (Figure 3D, insert; *t*_(8)_ = 2.94, *P* = 0.019). Furthermore compared to baseline, responses were significantly depressed (Figure 3E; 82.2 ± 3.8% baseline; *t*_(4)_ = 4.90, *P* = 0.016). Thus under otherwise subthreshold stimulation conditions, enhancement of the eCB system permits M_1_ mAChR LTD in mPFC neurons.

### M_1_ mAChR LTD is Recapitulated in Adult Mouse mPFC

**Figure 4:**
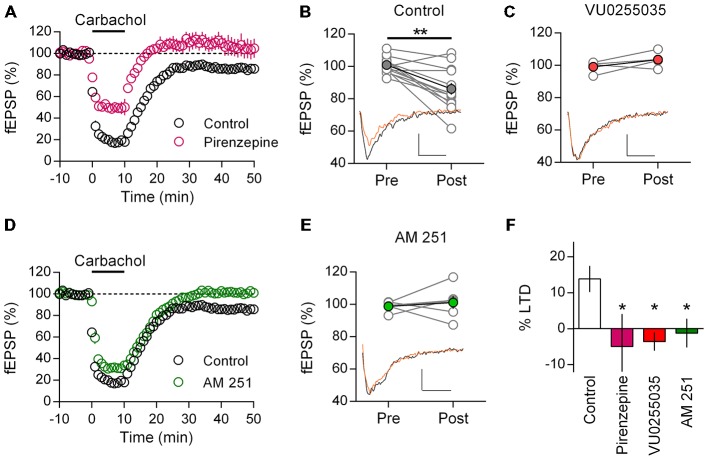
**Adult mice show M_1_ mAChR dependent LTD in mPFC mediated by CB_1_R. (A)** Time course of normalized field EPSPs in response to acute carbachol (10 μM, 10 min) stimulation in presence of M_1_ mAChR antagonist pirenzepine (0.5 μM, *n* = 6; control, *n* = 14). **(B)** Individual experiments (gray) and group average, pre (baseline) and post (40 min washout) carbachol treatment (**P < 0.01). Below, example traces pre (black) and post (orange) carbachol treatment (scale bar: 5 ms, 0.1 mV). **(C)** Similar plot with highly selective M_1_ mAChR antagonist VU0255035 treated group before (Pre) and after (Post) carbachol challenge; group averages in red (10 μM, *n* = 4). Below, example traces. **(D)** Time course showing normalized field EPSPs in response to carbachol stimulation in presence of CB_1_R antagonist AM251 (4 μM, *n* = 6). **(E)** Before-after carbachol challenge plot of individual experiments in presence of AM251, group average in green. Below, example traces. **(F)** Summary bar chart of percent LTD 40 min after carbachol washout (*P < 0.05).

Our findings and those of others suggest that M_1_ mAChR mediated LTD is a robust phenomenon found throughout development in the rat mPFC. However it is unclear if these findings can be generalized, especially in light of reported interspecies differences in cholinergic innervation and AChR synaptic modulation (Gil et al., 1997; Van der Zee and Keijser, 2011). Therefore we repeated our LTD experiments in the adult mouse mPFC. Recording layer 5 field potentials we challenged mPFC neurons with an identical 10 min carbachol (10 μM) protocol. Similar to the rat, carbachol induced a strong acute depression of field EPSPs, that after washout remain depressed and was absent in the presence of M_1_ mAChR antagonist pirenzepine (Figure 4A). Compared to baseline responses were significantly depressed 40 min after carbachol washout (86.1 ± 3.6%; *t*_(12)_ = 4.34, *P* = 0.001; Figure 4B). Furthermore in the presence of the highly selective M1 muscarinic receptor antagonist VU 0255,035 carbachol induced depression was also absent. Plotting individual experiments before and 40 min after carbachol washout in the presence of VU 0255,035, average responses were not different to baseline values (Figure 4C; 103.6 ± 2.6%, *n* = 4). Furthermore, compared to control experiments VU0255035 significantly interacted with the carbachol induced LTD (two-way repeat measure ANOVA *F*_(1,15)_ = 8.93, *P* = 0.009), due to an inhibition of the carbachol induced LTD (*P* < 0.01, Holm-Sidak’s multiple comparisons test). Thus a M_1_ mAChR mediated LTD is also found in the adult mouse mPFC.

Adopting the same approach, we tested for a CB_1_R dependent mechanism with AM251. Incubating mPFC neurons in AM251 did not affect the acute depression induced by carbachol, however after washout responses recovered to baseline values indicating an inhibition of LTD (Figure 4D). Comparing individual experiments, fEPSPs 40 min after carbachol challenge were unchanged from baseline (101.2 ± 4.0%, *n* = 6; Figure 4E). Furthermore, compared to control experiments, AM251 significantly interacted with the carbachol induced LTD (two-way repeat measure ANOVA *F*_(1,15)_ = 7.37, *P* = 0.016), due to an inhibition of the carbachol induced LTD (*P* < 0.01, Holm-Sidak’s multiple comparisons test). Expressing the LTD as a percent depression of baseline values, we compared the carbachol effect across treatments and identified a significant effect (one-way ANOVA *F*_(3,24)_ = 3.82, *P* = 0.023; Figure 4F). *Post hoc* analysis showed that treatment with either M_1_ mAChR antagonist or CB_1_R antagonist significantly reduced the induction of LTD compared to controls (Pirenzepine: *P* < 0.05; VU 0255, 035: *P* < 0.05; AM251: *P* < 0.05; Holm-Sidak’s multiple comparisons test). Therefore not only do mice share with rats an acetylcholine mediated form of LTD in the mPFC, but also the same M_1_ mAChR and CB_1_R dependent mechanism is engaged.

### Coupling Synaptic Activity with Endogenous Release of Acetylcholine Results in mPFC LTD

**Figure 5:**
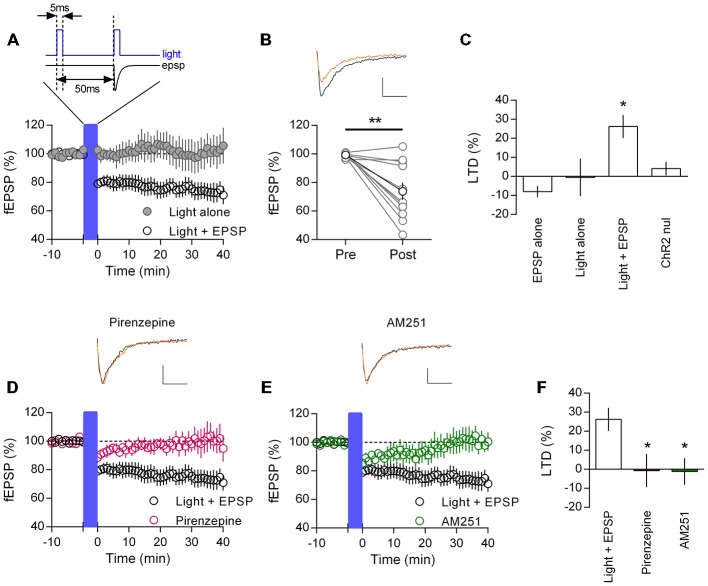
**Endogenously released acetylcholine coupled to excitatory synaptic activity induces CB_1_R mediated LTD in mouse mPFC. (A)** Time course of normalized field EPSPs in ChR2:ACh mouse challenged with a coupled light—EPSP 1 Hz stimulation train (1200 pulses (blue bar), protocol above). Coupled light and EPSP time course (clear circles, *n* = 11), light alone without EPSP time course (gray, *n* = 5). **(B)** Normalized field EPSPs from individual experiments at baseline (Pre) and 40 min after (Post) light—EPSP stimulation protocol, in black group average (**P < 0.01). Above: example traces pre (black) and post (orange) light—EPSP protocol (scale bar: 5 ms, 0.1 mV). **(C)** Summary plot of percent LTD calculated 40 min after end of stimulation protocol (*P < 0.05). **(D)** ChR2:ACh mouse field EPSP time course challenged with light—EPSP stimulation protocol (blue bar) in presence of M_1_ mAChR antagonist pirenzepine (0.5 μM, *n* = 5). Example traces above, pre (black) and post (orange) stimulation protocol. **(E)** Time course showing field EPSPs in response to light/EPSP stimulation protocol in ChR2:ACh mouse in presence of CB_1_R antagonist AM251 (4 μM, *n* = 6). **(F)** Summary bar chart of percent LTD 40 min after light—EPSP stimulation protocol.

Cholinergic afferents from the basal forebrain richly innervate the mPFC (Figure 1A). Therefore we asked if endogenous release of acetylcholine might also induce M_1_ mAChR mediated LTD. We adopted an optogenetic approach, using mice that selectively express channelrhodopsin in cholinergic neurons (ChR2:ACh mouse) and stimulated local acetylcholine release in the mPFC with a fiber optic. Previously, a 20 min electrical paired-pulse 1 Hz protocol has proven successful in inducing M_1_ mAChR LTD in juvenile animals (Volk et al., 2007; Huang and Hsu, 2010). We adapted the same protocol to our light induced acetylcholine release experiments, stimulating paired-pulse release (50 ms interval) at 1 Hz for 20 min. However we found that light induced acetylcholine release alone did not cause a depression in responses from layer 5 neurons (Figure 5A). Field EPSPs 40 min after the stimulation protocol were not different from baseline values (100.5 ± 9.9%, *n* = 5). A possible confounding issue in our light protocol is that compared to our carbachol protocol, there is no concurrent EPSP induction during the acetylcholine challenge. Hence, we wondered if coupling our light protocol with a single evoked EPSP timed to the second light pulse might permit LTD induction (Figure 5A, insert). We incubated our slices in the NMDAR antagonist D-AP5 (50 μM) to avoid any low frequency stimulation effects and repeated the 1 Hz paired-pulse protocol with a coincident EPSP. In this scenario we saw a strong depression of field EPSPs that persisted for the length of the recording (Figure 5A). On average responses as a percent of baseline were depressed to 75.3 ± 7.2% (*n* = 11) 40 min after the coupled light EPSP protocol. We analyzed individual experiments before and after the protocol and found a significant depression in responses (Figure 5B; *t*_(10)_ = 4.12, *P* = 0.002). Importantly when we repeated the same protocol without light induced acetylcholine release we failed to observe a depression of responses (fEPSP percent of baseline: 108.0 ± 3.0%, *n* = 3). We compared the percent LTD in the ChR2:ACh mouse with light and/or evoked EPSP with a control ChR2 null mouse challenged with the same light—EPSP protocol and identified a significant effect (Figure 5C; one-way ANOVA *F*_(3,20)_ = 4.73, *P* = 0.012). *Post hoc* analysis indicated this was due to a significant depression in ChR2:ACh mice with the coupled light—EPSP protocol that was not found in ChR2 null mice or in mice without coupled light and synaptic activity (*P* < 0.05, Holm-Sidak’s multiple comparisons test). Thus the induction of endogenous acetylcholine mediated LTD in the mPFC requires the coupling of acetylcholine release with a coincident post-synaptic potential.

To confirm the same M_1_ mAChR LTD mechanism was engaged with release of endogenous acetylcholine, we repeated experiments in the presence of the M_1_ mAChR antagonist pirenzepine. The coupled light and EPSP protocol resulted in a small acute depression in field EPSP responses, however fEPSPs subsequently recovered such that 40 min after the protocol responses had returned to baseline (Figure 5D; Percent of baseline, pirenzepine: 100.7 ± 8.7%, *n* = 5). Likewise we repeated experiments while antagonizing the CB_1_R with AM251. Again the coupled light and EPSP protocol induced a short depression of responses, however 40 min later responses recovered to baseline (Figure 5E; 101.1 ± 7.0%, *n* = 6). Expressing responses 40 min after the coupled light and EPSP protocol as a percent LTD, we compared layer 5 mPFC LTD inductions across treatments (Figure 5F). The mean LTD was different between groups (one-way ANOVA *F*_(2,19)_ = 5.44, *P* = 0.013). *Post hoc* analysis confirmed that the presence of M_1_ mAChR or CB_1_R antagonists significantly inhibited the induction of LTD (*P* < 0.05, Holm-Sidak’s multiple comparisons test). Therefore layer 5 neurons in the adult mPFC do expresses a M_1_ mAChR and CB_1_R mediated LTD in response to endogenous acetylcholine release, but only conditionally with a synchronous EPSP.

## Discussion

CB_1_R are highly expressed throughout the central nervous system, where they predominantly modulate neurotransmitter release at axonal terminals (Heifets and Castillo, 2009). Thus activating CB_1_Rs with endogenous or exogenous cannabinoids can dramatically affect synaptic function throughout the brain. In the PFC CB_1_Rs are found at both inhibitory and excitatory synapses and are able to modulate both short-term and long-term synaptic depression (Auclair et al., 2000; Fortin and Levine, 2007; Lafourcade et al., 2007; Chiu et al., 2010; Yoshino et al., 2011). Notably layer 5 pyramidal neurons express an mGluR5 dependent eCB mediated LTD in response to native firing patterns, that is sensitive to disruption in many neuropathological conditions (Lafourcade et al., 2007, 2011; Jung et al., 2012; Kasanetz et al., 2013; Lovelace et al., 2014; Thomazeau et al., 2014). This mGluR5 – CB_1_R LTD is particularly relevant to the M_1_ mAChR LTD described here, since both mGluR5 and M_1_ AChRs couple to the same G-protein class (G_q/11_), which via activation of PLC lead to the production of the eCB precursor diacylglycerol (Heifets and Castillo, 2009; Katona and Freund, 2012). Previous reports have linked activation of PLC to the induction of M_1_ mAChR mediated LTD in the mPFC (Huang and Hsu, 2010; Caruana et al., 2011; Ghoshal et al., 2015), however this is the first time a demonstration of a CB_1_R mediated mechanism has been made. Our findings suggest that activation of M_1_ mAChRs on layer 5 pyramidal neurons couples to local eCB mobilization leading to a CB_1_R mediated depression of glutamatergic neurotransmission.

Such a model is consistent with findings in both the hippocampus and striatum, where there is strong evidence of mAChR modulation of eCB signaling at inhibitory synapses. GABAergic synapses in the hippocampus and other brain areas express a short-term form of CB_1_R mediated synaptic plasticity termed depolarization-induced suppression of inhibition (DSI). Post-synaptic depolarization, either by experimenter induced voltage step or back-propagation of action potentials, engages a calcium dependent mobilization of eCBs that signal in a retrograde direction to presynaptic CB_1_Rs localized on GABAergic terminals (Kano et al., 2009). It is well established that low concentrations of carbachol potentiate DSI by enhancing eCB signaling independent of other mAChR ion channel-coupled mechanisms (Kim et al., 2002; Ohno-Shosaku et al., 2003; Narushima et al., 2007). Furthermore, engaging more vigorous DSI like protocols results in a CB_1_R mediated LTD at hippocampal inhibitory synapses (iLTD) that is also sensitive to mAChR activation (Younts et al., 2013). Equally at excitatory synapses that expresses eCB mediated LTD, evidence suggests that mAChR activation can either alone or with parallel synaptic activity induce CB_1_R mediated LTD (Colgin et al., 2003; Zhao and Tzounopoulos, 2011). Thus there is good evidence that the M_1_ mAChRs can engage the eCB system to induce CB_1_R mediated LTD.

Our experiments in both the adult rat and mouse mPFC suggest a robust engagement of the endocannbinoid system in response to direct pharmacological M_1_ mAChR activation and the induction of LTD. However these findings lie in contrast to a prior report studying M_1_ mAChR LTD in the juvenile mPFC, where the authors failed to find any CB_1_R dependance (Huang and Hsu, 2010). It is worth noting that there appear to be other differences present between juvenile and adult rats in the induction of M_1_ mAChR LTD. Experiments in the juvenile mPFC suggest a lower sensitivity to carbachol challenge (Huang and Hsu, 2010). Furthermore at this developmental age the magnitude of M_1_ mAChR LTD is insensitive to the duration of carbachol challenge (Caruana et al., 2011), in contrast to the clear time—response dependency found here. Of relevance, the engagement of eCB mediated synaptic modulation appears to be developmentally regulated (Castillo et al., 2012). DSI at hippocampal synapses is absent at early postnatal periods in the rat and importantly synergy between DSI and mGluR activation only becomes significant during adolescence (Zhu and Lovinger, 2010; Liang et al., 2014). Relative to other brain areas the mPFC shows a delayed developmental trajectory and it is postulated that a critical window to synaptic plasticity opens only upon reaching adolescence in this brain region (Selemon, 2013). Thus it is possible that mAChR induced eCB mobilization is only present at the later developmental stages studied here.

In our hands, depression of glutamatergic synaptic transmission in the mPFC with carbachol challenge proceeds in two phases; an initial acute depression of transmission presumably mediated by presynaptic M_2_/M_4_ mAChR (Lucas-Meunier et al., 2003), followed by an extended LTD after wash off. Brief periods of carbachol challenge (5 min) that nonetheless resulted in acute synaptic depression, failed to induce mAChR LTD, whereas longer periods of carbachol challenge resulted in a robust LTD. This is directly comparable to stimulus evoked LTD, where a 10 min 10 Hz protocol induces a robust CB_1_R mediated LTD, but a 5 min protocol is subthreshold (Lafourcade et al., 2007; Marrs et al., 2010). Importantly both subthreshold stimulus evoked LTD and subthreshold mAChR LTD can be converted to LTD by reducing hydrolysis of the eCB 2-AG with inhibition of MAGL. This is consistent with the idea that mAChR LTD in the mPFC proceeds via 2-AG mediated mechanism, wherein post-synaptic synthesis of 2-AG signals to pre-synaptic CB_1_R to induce LTD.

Cholinergic fibers projecting from the basal forebrain richly innervate the mPFC (Houser et al., 1985), however their long trajectory and mixing with both GABAergic and glutamatergic fibers makes selective electrical stimulation of these inputs difficult in the slice. Therefore we pursued an optogenetic approach and used mice selectively expressing channelrhodopsin in cholinergic neurons and induced local mPFC acetylcholine release via a fiber optic. Such an approach has recently proven successful in the hippocampus where selective electrical activation of cholinergic fibers is also problematic (Alger et al., 2014). We stimulated cholinergic afferents with brief light pulses at a 20 Hz interval, similar to reported native beta oscillations found in the basal forebrain (Quinn et al., 2010; Tingley et al., 2015). Nevertheless, initial attempts to induce mAChR LTD with local acetylcholine release in isolation were unsuccessful. However, when we coupled phasic acetylcholine release with an EPSP, CB_1_R dependent LTD was observed. Neither EPSP induction alone nor repeating the protocol in channelrhodopsin null animals resulted in LTD. Thus the release of endogenous acetylcholine can induce LTD, but requisite of cooperative synaptic activity.

This requirement for concomitant glutamatergic synaptic and M_1_ mAChR activity likely reflects the subtle composition of the eCB system and its suitability to signal integration. With supra-threshold stimulation either G_q/11_ coupled metabotropic receptors or post-synaptic calcium entry alone can induce eCB mobilization and synaptic depression, however by coupling subthreshold stimulations eCB LTD may equally be induced (Ohno-Shosaku and Kano, 2014). Notably in pyramidal neurons in the mPFC, M_1_ mAChR are enriched in spines, placing them well to coordinate synaptic activity (Yamasaki et al., 2010). Furthermore, the proposed coincidence detector molecule, PLC (Hashimotodani et al., 2005), appears robustly activated in the mPFC during M_1_ mAChR challenge (Huang and Hsu, 2010; Caruana et al., 2011). Regardless of the precise mechanism our results suggest that endogenously released acetylcholine results in LTD only in neurons where there is corresponding glutamatergic synaptic activity.

How this form of LTD correlates with mPFC dependent learning and function is speculative. Cholinergic input to the mPFC has a prominent role in promoting attention via modulation of local cortical networks (Hasselmo and Sarter, 2011; Klinkenberg et al., 2011; Bloem et al., 2014), and the eCB system has been proposed to modulate associated cortical oscillations (Alger et al., 2014). However more specifically, there is accumulating evidence that cholinergic afferents transmit salient information to the neocortex, which may direct specific behaviors in the mPFC (Parikh et al., 2007; Lin and Nicolelis, 2008; Tingley et al., 2015). The eCB system seems well positioned to integrate glutamatergic and cholinergic neurotransmission at layer 5 glutamatergic synapses, be it a reflection of general cortical arousal or specific acetylcholine release. In this context it is interesting to note that modulation of the eCB system is a developing target for the treatment of Alzheimer’s disease, where enhancement of cholinergic signaling has long been a principal therapeutic (Aso and Ferrer, 2014). Thus it may be that targeting of the eCB system could compensate for deficits in cholinergic signaling found in this disorder.

## Author Contributions

HM, AB, ALP and OJM designed research; HM, AB, CB and OL performed research; HM, AB and CB analyzed data; CB contributed regent and methods, HM and OJM wrote the paper; OJM supervised the entire project.

## Funding

INSERM, ANR CYFIP-Aut and Foundation Jêrome Lejeune (H.M.) supported this work.

## Conflict of Interest Statement

The authors declare that the research was conducted in the absence of any commercial or financial relationships that could be construed as a potential conflict of interest.
